# Autochthonous Mouse Melanoma and Mammary Tumors do not Express the Pluripotency Genes Oct4 and Nanog

**DOI:** 10.1371/journal.pone.0057465

**Published:** 2013-02-28

**Authors:** Caroline Schreiber, Vanessa Kuch, Viktor Umansky, Jonathan P. Sleeman

**Affiliations:** 1 Centre for Biomedicine and Medical Technology Mannheim (CBTM), Medical Faculty Mannheim, University Heidelberg, Mannheim, Germany; 2 Institute for Toxicology and Genetics, KIT Campus Nord, Karlsruhe, Germany; 3 Skin Cancer Unit, German Cancer Research Center, Heidelberg and Department of Dermatology, Venereology and Allergology, University Medical Center Mannheim, Ruprecht-Karl University of Heidelberg, Mannheim, Germany; University of Nevada School of Medicine, United States of America

## Abstract

The homeodomain transcription factors Oct4 and Nanog maintain pluripotency and self-renewal in embryonic stem cells. In somatic cells, inappropriate expression of these genes has been associated with loss of differentiation, malignant transformation, and the acquisition of cancer stem cell-like properties. As cancer stem cells have been suggested to underlie the growth and malignancy of tumors, Oct4 and Nanog may represent therapeutic targets. Their expression could also act as a marker of the cancer stem cell population, permitting its isolation and characterisation. Nevertheless, the existence of multiple pseudogenes and isoforms of these genes has complicated the interpretation of the data that supports a role for Oct4 and Nanog in the cancer context. Here we addressed this issue using knockin mice in which IRES elements are used to allow GFP expression under the control of the endogenous Oct4 or Nanog promoters, while maintaining correct expression of the Oct4 or Nanog gene. These mice were crossed with MT/ret mice that develop melanomas, and with MMTV-PyMT mice and MMTV-Neu mice that develop mammary adenocarcinomas. We analysed the tumors that developed in these compound mice for GFP expression. In this way we could assess transcription of Oct4 and Nanog in autochthonous cancers without the complication of factors such as pseudogene expression, alternative splicing and antibody specificity. Both the Oct4 and Nanog knockin tumor-bearing mice expressed GFP in blastocysts and testes as expected. However, we could find no evidence for expression of the GFP reporter above background levels in tumors using FACS, qPCR and immunohistochemistry. Furthermore, cultivation of Oct4GFP and NanogGFP MMTV-PyMT tumor cells either adherently or as spheroids had no effect on the expression of the GFP reporter. Together these data suggest that Oct4 and Nanog are not expressed in tumor cells that arise in the autochthonous cancer models studied here.

## Introduction

The idea that cancer is driven by a subpopulation of tumor cells with stem cell properties was proposed around 150 years ago [1]. During the last decade, the concept that tumor cells are organized in such a hierarchical manner has received increasing attention, and evidence for the existence of cancer stem cells (CSCs) has been garnered for both hematopoietic tumors and a variety of solid tumors, including breast, brain, prostate, colon and lung [2]. By definition, cancer stem cells are a subset of tumor cells that are able to self-renew, give rise to heterogeneous progeny, and initiate the growth of new tumors [3]. Stem cell properties such as indefinite growth, growth under non-adherent conditions, drug resistance and asymmetrical division have been attributed to CSCs [4]. These characteristics suggest that targeting of CSCs should be an effective anti-cancer strategy [2], [5]. Nevertheless, difficulties in isolating CSCs to study their properties have hampered progress in this area. Current marker-based strategies only enrich for CSC subpopulations [5]. Assays used in vitro to study CSCs do not reliably reflect tumor-initiating properties in vivo [6]. There is also increasing evidence that CSC properties are plastic and can be gained or lost, for example in response to microenvironmental cues [5]. Thus new ways of reliably identifying CSCs to facilitate their isolation and characterisation are required.

Similarities between embryogenesis and tumorigenesis have long been recognized [7], [8], [9]. Key embryonic pathways that regulate self-renewal and differentiation are frequently deregulated during tumor progression [7], [8]. Moreover, unlimited proliferation capacity and self-renewal are typical embryonic stem (ES) cell properties but are also attributed to tumor cells. In ES cells, self-renewal is maintained by the master regulators of pluripotency Oct4, Nanog and Sox2. Oct4 and Nanog are homeodomain transcription factors that are essential for the maintenance of pluripotency and early embryo development. They are highly expressed in pluripotent ES cells and down-regulated upon differentiation [10], [11], [12].

In the adult, Oct4 and Nanog expression is normally restricted to the ovary and testis [10], [11]. Ectopic expression of Oct4 or Nanog in somatic cells results in dedifferentiation, malignant transformation and causes dysplasia in epithelial tissue, demonstrating the oncogenic potential of pluripotency genes [13], [14], [15], [16]. Furthermore, ectopic expression of Oct4 in tumor cells results in dedifferentiation and enhanced CSC-like properties such as sphere formation, drug resistance and increased tumorigenicity [17], [18], [19]. Consistently, elevated expression of Oct4 and Nanog has been reported in cancer cell lines and/or primary cancers from melanoma [19], [20], germ cell tumors [21], [22], [23], [24], [25] and cancers of the breast [24], [26], [27], prostate [28], lung [29], colorectum [30] and endometrium [31], and correlates with increased malignancy and acquisition of CSC properties.

Based on these and other observations it has been suggested that Oct4 and Nanog are key factors in the induction and maintenance of CSC identity, for example through the regulation of self-renewal properties, and therefore represent potential therapeutic targets that could serve as markers of tumor cells with CSC properties [32], [33], [34]. Nevertheless, numerous pseudogenes and isoforms of both Oct4 and Nanog exist, which complicates the interpretation of studies that suggest that these genes determine the stemness of CSCs [35], [36]. Furthermore, concerns have been raised about the reliability of antibodies used in some of these studies [35], [37], [38].

Here we have addressed the issue of whether Oct4 and/or Nanog are expressed in oncogene-driven spontaneous tumors arising in transgenic mouse cancer models. To this end we used “knockin” transgenic mice that express GFP under the control of the endogenous Oct4 or Nanog promoters [39], [40]. These mice were crossed with MT/ret mice that develop melanocytic tumors in response to transgenic expression of the Ret oncogene [41], with MMTV-PyMT transgenic mice that develop polyomavirus middle T oncogene-driven multifocal mammary adenocarcinomas [42], and with MMTV-Neu mice that develop polyclonal mammary adenocarcinomas due to expression of the activated Neu oncogene in the mammary epithelium [43]. Tumors developing in these compound transgenic mice were analysed for expression of the GFP reporter. This approach allowed us to examine the transcriptional expression of Oct4 and Nanog in autochthonous tumors while avoiding problems associated with pseudogene expression, alternative isoforms and antibody specificity. Furthermore, the GFP reporter gave us the possibility of using FACS sorting to isolate Oct4- or Nanog-expressing tumor cells to examine their CSC properties. We found that although expression of the GFP reporter could be readily detected in blastocysts and testes from these mouse lines as expected, no GFP expression above background levels could be detected in the tumors and tumor cells derived from the animals. These data therefore suggest that transcription of Oct4 and Nanog is unlikely to be a key determinant of CSC properties in these autochthonous tumor models.

## Materials and Methods

### Ethics

All mice were maintained under specific pathogen-free conditions in accordance with German government and institute guidelines and regulations. The protocol was approved by the Institutional Animal Care and Use Committee of the Karlsruhe Institute of Technology (KIT) and permission was granted by the Regierungspräsidium Karlsruhe.

### Mice

Tg(Nanog–GFP, puro)1Yam (Nanog-GFP) mice were obtained from the RIKEN BioresourceCenter and genotyped as described [40]. The B6;129S4-^Pou5f1tm2Jae^/J (Oct4-GFP) mice were obtained from the Jackson Laboratories and genotyping was performed as described [39]. Both Nanog-GFP and Oct4-GFP mice were crossed onto the FVB background for at least six generations before they were crossed with FVB; MMTV-PyMT and FVB; MMTV-Neu mice. The MT/ret mice [41] were held on a mixed background. Mice were monitored daily. Tumors from Oct4GFP or NanogGFP transgenic MMTV-Neu and MMTV-PyMT mice were analysed before the tumor size reached 1 cm in diameter in one direction. Analysis of compound MT/ret tumors was performed before the tumors reached 0.6 cm in diameter.

### Flow Cytometry

Testes were prepared from control or compound MT/ret mice and passed through a 40 µm filter to obtain a single cell suspension. MT/ret control and compound tumors were minced and digested using collagenase II/collagenase IV (Invitrogen) and DNaseI (Applichem) for 40 min at 37°C. After lysis of red blood cells, cells were passed through a 40 µm filter to obtain a single cell suspension. Mammary tumors from control and compound mice were minced, washed in PBS and digested for 1 h at 37°C with collagenase/hyaluronidase (Stem Cell Technologies). Samples were subsequently digested with trypsin/EDTA for 2 min and with dispase (Sigma)/DNaseI (Applichem) for 10 min at 37°C. After lysis of red blood cells, cells were passed through a 40 µm filter and enriched for Lin- cells using FACS sorting to exclude CD45.2, Ter119, CD31 and CD140a-positive cells. FACS analysis of single cell suspensions from either testes, melanomas or mammary tumors was then performed using a FACSCantoII (BD Biosciences) to determine GFP expression. Dead cells were excluded using SytoxRed. Cells from NanogGFP+ or Oct4GFP+ testes were used as positive controls. Cells from MT/ret, MMTV-PyMT and MMTV-Neu control tumors served as negative controls.

### RNA Isolation and Realtime-qPCR

RNA of testes, control and compound tumors was isolated using Trizol (Invitrogen) according to the manufacturer’s protocol. Subsequently, 5 µg of RNA was digested by 5U DNaseI (Thermo Fisher Scientific) for 30 min at 37°C. The reaction was stopped by addition of EDTA and heat inactivation, and RNA was precipitated using isopropanol. DNA-free RNA was transcribed into cDNA using reverse transcriptase (Thermo Fisher Scientific) according to manufacturer’s protocol. GFP, Oct4 and Nanog expression was analysed using SYBR-Green mix (Applied Biosciences) to perform realtime qPCR using the OneStepPlus RealtimePCR System (Applied Biosciences) under the following PCR conditions: 15 sec 95°C, 1 min 60°C, 1 min 72°C. The ribosomal protein PO was used as a control to normalize the data [44]. The following primer pairs were used to amplify the indicated cDNAs:

Oct4: (Oct4-for 5′- AGCACGAGTGGAAAGCAACT-3′ and Oct4-rev 5′- TTCTAGCTCCTTCTGCAGGG -3′).

Nanog: (Nanog-for 5′- AACCAAAGGATGAAGTGCAAGCGG - 3′ and Nanog-rev 5′- TCCAAGTTGGGTTGGTCCAAGTCT -3′).

EGFP: (EGFP-for 5′- GTTGGAGAAGGTGGAACCAACTC -3′ and EGFP-rev 5′- AGGGTGTCGCCCTCGAA - 3′).

Ribosomal protein PO: (RibPo-for 5′-GGACCCGAGAAGACCTCCTT-3′ and RibPo-rev 5′- GCACATCACTCAGAATTTCAATGG-3′).

### Immunofluorescence

Testes, control and compound tumors were fixed in 4% PFA/10% sucrose at 4°C overnight and embedded in OCT (TissueTEK). For analysis, tumors were cut into 8 µm sections, with 10 to 15 planes per tumor, and each plane 24 µm apart. Frozen sections were dried overnight and immunofluorescence was performed the next day as follows. Sections were permeabilized with 0.1% TritonX/0.3M glycine, blocked with 5% BSA, then incubated with anti-GFP antibody (Living Colors A.V. Peptide antibody, Clontech) at 4°C overnight. Sections were washed in PBS, then incubated with anti-rabbit Alexa-488 secondary antibody (Invitrogen) for 1 h at RT. Nuclei were stained with DAPI. To reduce autofluorescence background, sections were incubated with 3% Sudan black (Roth) in 70% EtOH for 5 min, followed by two PBS washes, one in 70% EtOH and two in PBS. Sections were mounted and analysed using an AxioVert Zeiss microscope.

### Tissue Culture and Sphere Formation

Cells from MMTV-PyMT and MMTV-Neu control and GFP compound tumors were isolated as described above. Lin- cells were enriched using MACS sorting to exclude CD45, Ter119, CD31 and CD140a-positive cells, and were then plated either as monolayer cultures in DMEM/10% FCS or as suspension spheres as described previously [6]. RNA was isolated from adherently cultured cells and from spheroids, and qPCR analysis was performed as described above.

## Results

Tg(Nanog-GFP, Puro)1Yam (NanogGFP) mice express GFP under the control of the endogenous Nanog promoter due to integration into the 5′ untranslated region of the GFP gene coupled to an IRES element [40]. Similarly, B6;129S4-Pou5f1^tm2Jae/J^(Oct4GFP) mice express GFP under the control of the endogenous Oct4 promoter due to integration of the GFP gene coupled to an IRES element between the Oct4 stop codon and the polyadenylation signal [39]. These mice were crossed with MT/ret [41], MMTV-PyMT [42] and MMTV-Neu [43] transgenic mice to generate compound mice that spontaneously developed melanomas or mammary tumors, respectively, and that also expressed GFP under the control of the endogenous Nanog or Oct4 promoter.

**Figure 1:**
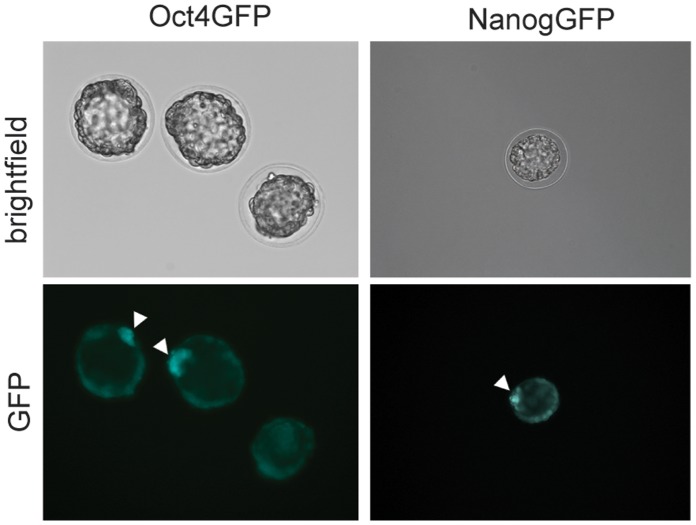
Oct4GFP and NanogGFP expression in inner cell mass of transgenic mouse embryos. Blastocysts were isolated from pregnant Oct4GFP+ (left side) or NanogGFP+ (right side) mice. Bright field and fluorescence pictures were taken. Arrows indicate GFP+ cells in the inner cell mass (ICM) of the blastocysts.

**Figure 2:**
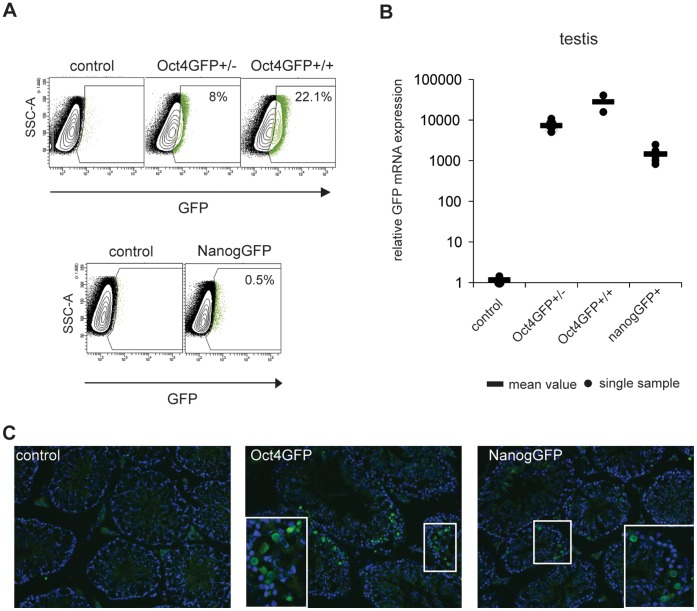
Oct4GFP and NanogGFP are expressed in mouse testes. (A) Cells from testes of GFP−/− control transgenic mice, Oct4GFP+/−, Oct4GFP+/+ and NanogGFP+ animals were isolated and analysed for GFP positivity by FACS. Representative flow cytometry analyses in which sideward scatter is plotted against GFP fluorescence are shown for each genotype. The percentage of GFP+ cells compared to GFP negative control testes is indicated in the gate. (B) GFP mRNA expression in control (n = 3), Oct4GFP+/− (n = 6), Oct4GFP+/+ (n = 2) and NanogGFP+ (n = 5) testes was analysed by qPCR. Circles represent individual samples, the bar indicates the mean value of all samples. (C) GFP+ cells can be detected in Oct4GFP+ and NanogGFP+ testes but not in control testis. Representative pictures of immunofluorescence staining with anti-GFP antibody are shown. Magnification 200×. The framed region is enlarged in the insert.

First we verified that the GFP reporter is expressed appropriately in these mice. As expected, GFP expression could be readily observed in the inner cell mass of transgenic blastocysts (Figure 1). Furthermore, we examined GFP expression in the testis as Oct4 and Nanog are expressed in spermatogonia [45], [46]. Using FACS analysis, GFP-positive cells were found to be present in the testes from both Oct4GFP and NanogGFP compound mice (Figure 2A). Furthermore, GFP mRNA expression could be readily detected in testis by qPCR analysis (Figure 2B), and single GFP positive cells were observed in testis sections using immunofluorescence staining, consistent with the expression of Oct4 and Nanog in early type-A spermatogonia (Figure 2C).

**Figure 3:**
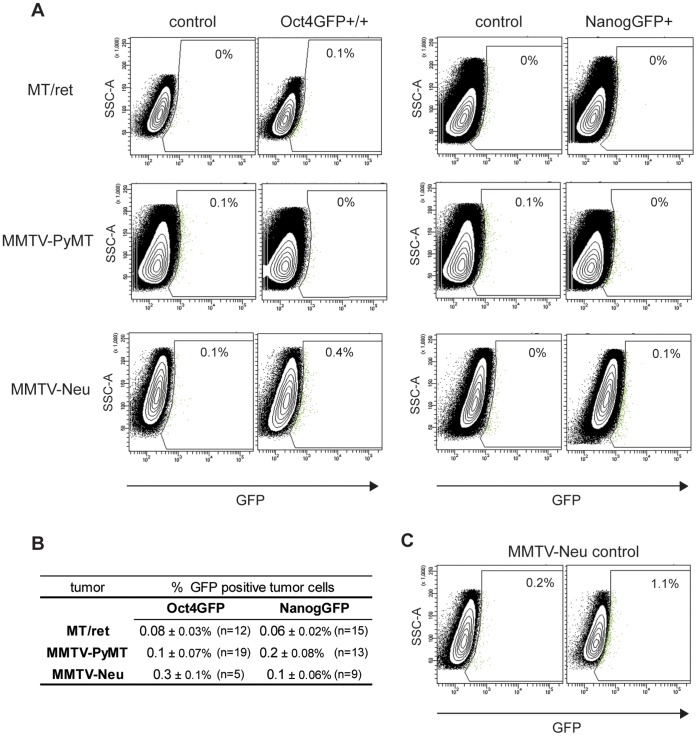
No GFP+ subpopulation can be detected in MT/ret, MMTV-PyMT or MMTV-Neu compound tumors. (A) Representative flow cytometry analyses in which sideward scatter is plotted against GFP fluorescence are shown for Oct4GFP+ or NanogGFP+ MT/ret, MMTV-PyMT and MMTV-Neu tumor cells and their respective GFP−/− control tumor cells. Cells were freshly isolated from tumors, enriched for Lin- cells and analysed for GFP expression by FACS. The percentage of GFP+ cells compared to GFP negative control tumors is indicated in the gate. (B) Table summarizing the results of the FACS analyses performed. The numbers represent the mean value of the percentage of GFP+ cells of all analysed samples per tumor model, ± SEM. The total number of analysed samples per tumor model is indicated in brackets. (C) Representative FACS analysis of two Neu control tumors showing variation in background fluorescence.

Having established that GFP is expressed appropriately under the control of the endogenous Oct4 or Nanog promoters in the compound transgenic mice, we then used FACS analysis to investigate the presence of GFP+ tumor cells in primary tumors from Oct4GFP and NanogGFP MT/ret, MMTV-PyMT and MMTV-Neu mice. As a negative control, tumors from MT/ret, MMTV-PyMT and MMTV-Neu without a germline GFP knockin in the Nanog or Oct4 genes were used. Disaggregated tumor cells were stained with CD31, CD45.2, CD140a and Ter-119 to allow leukocytes and endothelial, erythroid and mesenchymal cells to be excluded from the analysis. Dead cells were also excluded. A gate for GFP-positive cells was set using a control tumor, and all positive events in this gate for the corresponding knockin tumor cells and additional negative control tumor cells were measured (Figure 3). We could not detect a GFP+ subpopulation of tumor cells for any of the analysed knockin tumors. Although for some mice we observed an apparent very low percentage of GFP+ tumor cells above background (Figure 3A and B), we also observed a similar distribution of GFP+ cells in the negative controls in which no GFP could be expressed (Figure 3A and 3C), suggesting that the very low percentage of apparently GFP-positive tumor cells from the Oct4GFP and NanogGFP tumors reflects variation in the background signal in the gated GFP channel.

**Figure 4:**
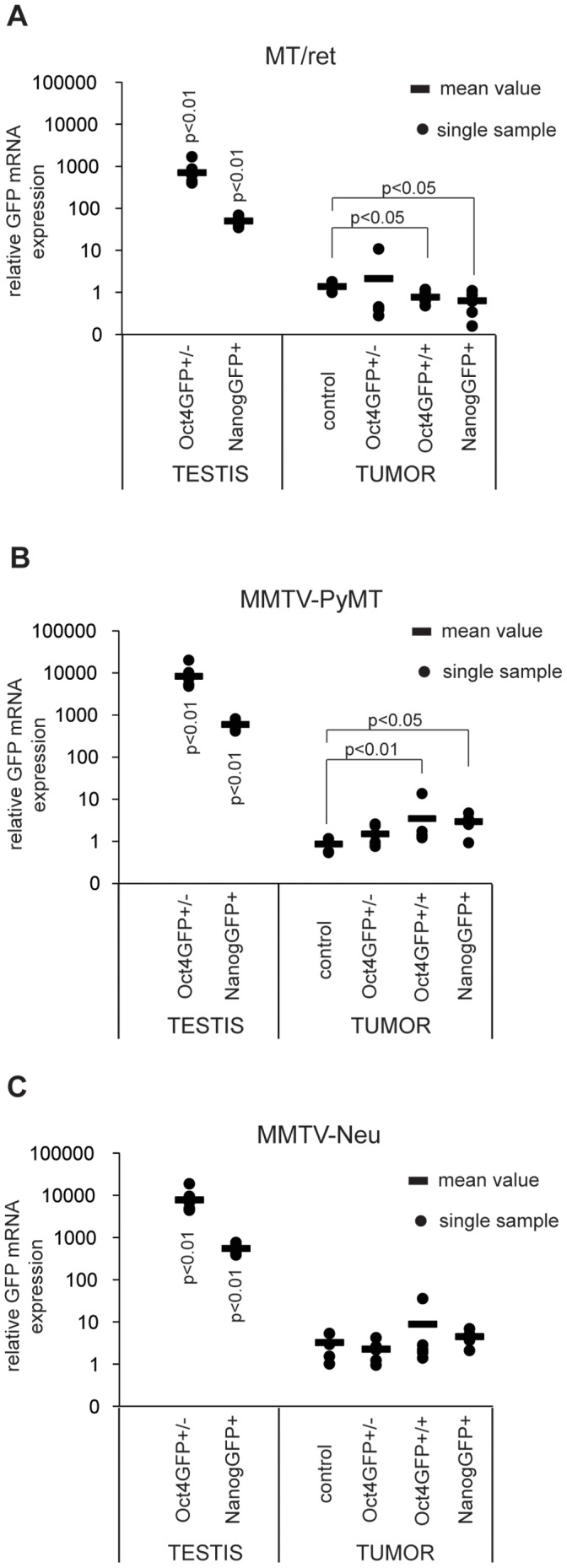
GFP mRNA is not expressed in Oct4GFP and NanogGFP transgenic MT/ret, MMTV-PyMT or MMTV-Neu tumors. Relative GFP mRNA expression levels of (A) MT/ret, (B) MMTV-PyMT and (C) MMTV-Neu compound tumors with indicated genotypes and Oct4GFP+ and NanogGFP+ testes were analysed by qPCR. For each tumor model, GFP mRNA expression levels of compound tumors and Oct4GFP+ and NanogGFP+ testes were compared to one control tumor which was set to 1. Circles represent individual samples, the bar indicates the mean value of all samples. The number of analysed samples is as follows: testes: Oct4GFP+/− n = 7, NanogGFP+ n = 5. (A) MT/ret: control n = 4, Oct4GFP+/− n = 6, Oct4GFP+/+ n = 6, NanogGFP+ n = 6 (B) MMTV-PyMT: control n = 5, Oct4GFP+/− n = 6, Oct4GFP+/+ n = 6, NanogGFP+ n = 6 (C) MMTV-Neu: control n = 5, Oct4GFP+/− n = 5, Oct4GFP+/+ n = 5, NanogGFP+ n = 9.

To further examine whether or not GFP-positive cells were present in the GFP-knockin tumors, we analysed GFP mRNA expression in the tumors using qPCR. In comparison to GFP expression in testes from Oct4GFP and NanogGFP mice, expression levels in the Oct4GFP+ and NanogGFP+ tumors was 100–10 000 times lower depending on the analysed tumor model (Figure 4). Furthermore, no significant increase in GFP expression compared to GFP-negative control tumors could be detected in either the Oct4GFP+ and NanogGFP+ MT/ret and MMTV-Neu tumors. Indeed, in the case of the melanomas a significant decrease in GFP levels was observed in the Oct4GFP+ and NanogGFP+ tumors compared to the negative controls (Figure 4A), indicating that the expression of GFP transcription was below the threshold of detectability. For the MMTV-PyMT tumors, a three-fold significant increase in GFP expression was observed in the Oct4GFP+ and NanogGFP+ tumors compared to GFP negative tumors (Figure 4B), albeit from a very low basal level close to the noise threshold. Together these data suggest again that very few if any GFP-expressing cells were present in the Oct4GFP+ and NanogGFP+ tumors.

**Figure 5:**
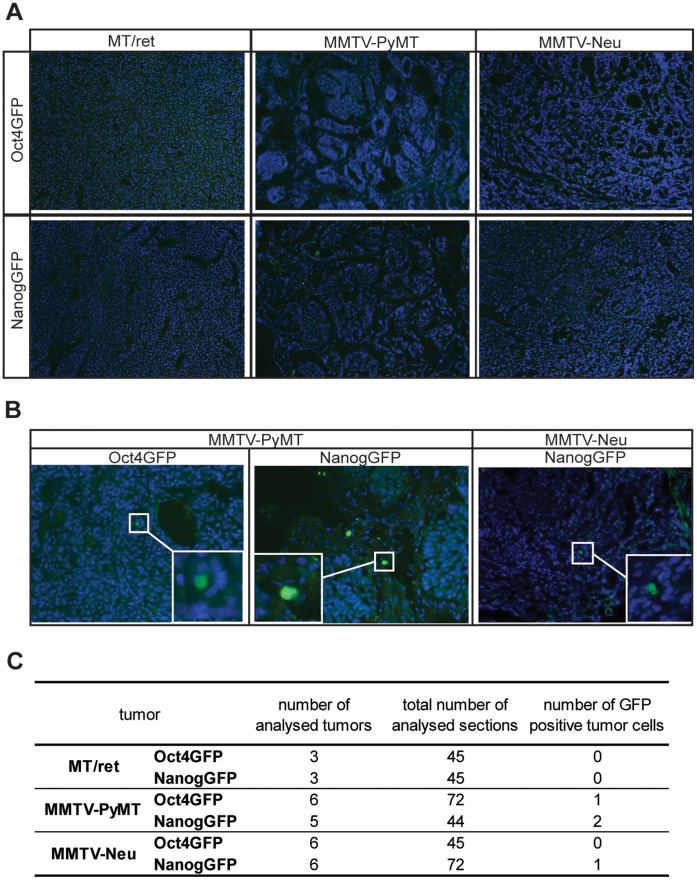
Only very rare GFP+ cells can be detected in compound MMTV-PyMT or MMTV-Neu tumors. Sections of Oct4GFP+ or NanogGFP+ MT/ret, MMTV-PyMT and MMTV-Neu tumors were stained with an anti-GFP antibody and analysed for GFP positive cells. (A) Representative pictures of GFP immunofluorescence stainings showing the GFP negativity of virtually all sections from the compound tumors. (B) Pictures of the only GFP+ cells detected in single sections from Oct4GFP+ and NanogGFP+ MMTV-PyMT and MMTV-Neu tumors. The framed region is enlarged in the insert. (C) Table summarizing the immunofluorescence analysis, showing the number of analysed tumors per tumor model, the total number of analysed sections, and the total number of detected GFP+ cells per tumor model.

Finally we examined GFP expression in tumor sections. No direct GFP signal could be observed in sections from Oct4GFP+ and NanogGFP+ tumors (data not shown). We therefore stained the sections with anti-GFP antibody in an attempt to increase the sensitivity of detection. Tumors from between 3 to 6 animals were analysed per animal model, with at least 45 sections per tumor type analysed in total. In MT/ret primary tumors no NanogGFP or Oct4GFP positive cells could be detected (Figure 5A and C). The vast majority of analysed sections of MMTV-PyMT and MMTV-Neu Oct4GFP+ and NanogGFP+ tumors were also negative. However, a single Oct4GFP-positive cell and two NanogGFP-positive cells were detected in single sections from MMTV-PyMT tumors (Figure 5B and C). A single NanogGFP positive cell was also detected in a section from one MMTV-Neu tumor. These data again suggest that Oct4 and Nanog were expressed at virtually non-detectable levels in the MT/ret melanoma and MMTV-PyMT and MMTV-Neu mammary tumor cells.

**Figure 6:**
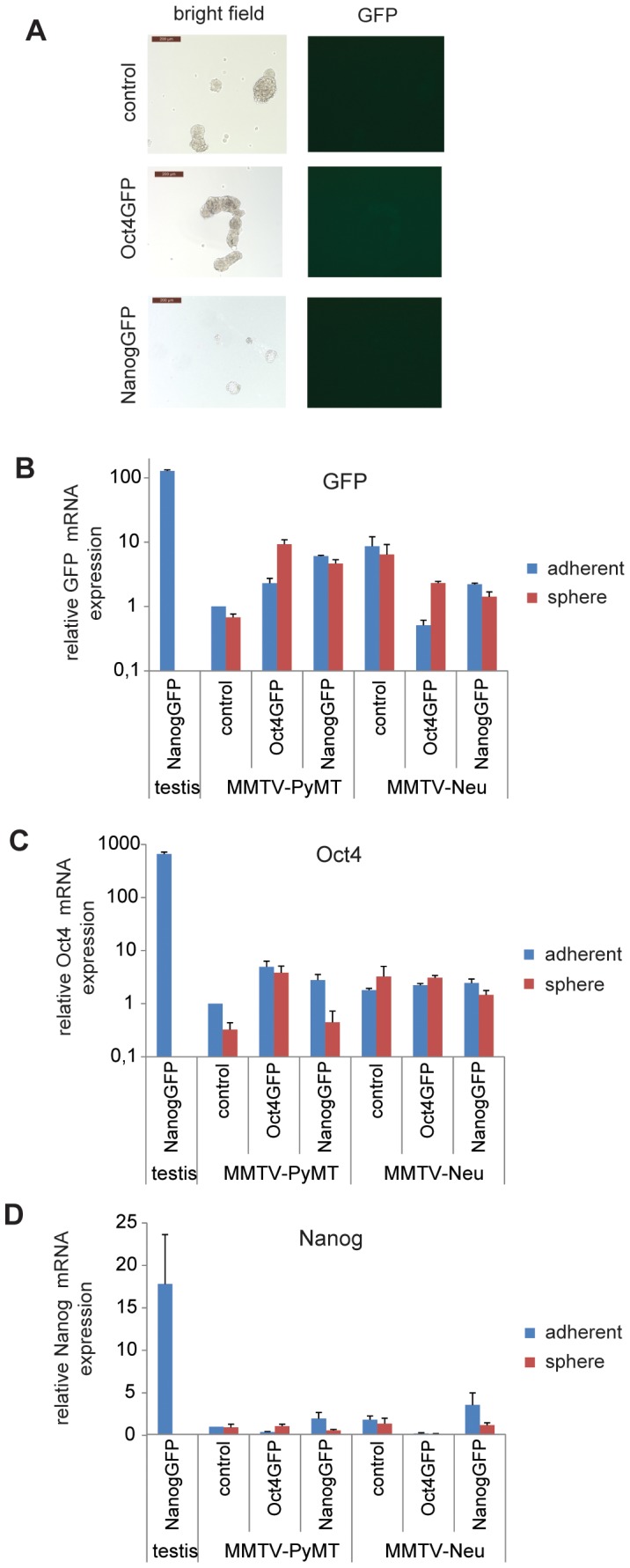
Spheroid cell culture does not induce Oct4GFP or NanogGFP expression in MMTV-PyMT or MMTV-Neu tumor cells. (A) No GFP+ cells can be detected in spheroids of Oct4GFP+ or NanogGFP+ MMTV-PyMT tumor cells. Oct4GFP+ or NanogGFP+ MMTV-PyMT tumor cells were freshly isolated from tumors, grown as spheres for one passage when pictures were taken. Representative bright field (left side) and fluorescent (right side) pictures are shown. Scale bar = 200 µm. (B-D) Freshly isolated Oct4GFP+ or NanogGFP+ MMTV-PyMT and MMTV-Neu tumor cells were either grown adherently (passage 1–4) or as spheres (passage 0) and analysed for relative expression of (B) GFP, (C) Oct4 and (D) Nanog mRNA by qPCR. mRNA expression of NanogGFP+ testis and compound MMTV-PyMT and MMTV-Neu tumor cells was compared to mRNA expression of control MMTV-PyMT tumor cells, which was set to 1. The analysis was performed in triplicate. The mean ± SEM is shown.

Non-adherent culture of tumor cells as spheres has been reported to enrich for CSCs and to increase the expression of genes such as Oct4 and Nanog [20], [47], [48]. We therefore freshly isolated cells from Oct4GFP+ and NanogGFP+ MMTV-PyMT or MMTV-Neu tumors and plated them either adherently or as suspension spheres. Bright field and fluorescent pictures of the spheres were taken, then GFP expression was determined by qPCR analysis. No GFP+ cells were detected using fluorescent microscopy in spheres derived from Oct4GFP+ and NanogGFP+ MMTV-PyMT or MMTV-Neu tumors (Figure 6A and data not shown). Furthermore, no increase in expression of GFP, Oct4 or Nanog was observed in qPCR analysis of either adherent or spheroid-cultured Oct4GFP+ and NanogGFP+ cells (Figure 6B–D). Together the data suggest that cultivation of tumor cells from the autochthonous cancer models studied here, even under conditions that might be expected to increase CSC numbers, does not result in enhanced expression of Oct4 or Nanog.

## Discussion

Oct4 and Nanog have been suggested to endow tumor cells with stemness properties such as self-renewal, and to thereby regulate the CSC compartment in tumors. However, a number of caveats are associated with this notion. The idea that Oct4 confers stemness properties is also controversial as it is dispensable for the maintenance of somatic stem cells [39]. The data we present here using autochthonous murine melanoma and breast cancers strongly suggests that Oct4 and Nanog are not expressed in these tumors.

A number of factors complicate the analysis of Oct4 and Nanog, potentially leading to inappropriate conclusions. Oct4 is extensively alternatively spliced [35], [49]. The main splice variants Oct4A and Oct4B differ at their N-terminus as exon 1 is missing in the Oct4B variant and has instead additional sequence from the intron 1–2 region. Oct4A and Oct4B have different subcellular locations and different functions [35]. Antibodies whose epitope is located in the C-terminus of the protein cannot therefore distinguish between these two isoforms. Commonly-used microarrays for transcriptome analysis also do not distinguish between Oct4A and Oct4B [50]. Importantly, only the Oct4A isoform is located in the nucleus and regulates self-renewal [51]. Although expression of Oct4A has been reported in a subpopulation of prostate cancer cells, the protein was cytoplasmically located [52], raising the question as to whether this observation is relevant for the CSC properties of the tumor cells. The GFP reporter knockin approach we used here would reflect transcription of both Oct4 isoforms. However, no transcription was detected in our experiments. Consistently, Oct4A is not expressed in HeLa and MCF7 cell lines due to promoter methylation [53].

Numerous transcribed pseudogenes exist for both Oct4 and Nanog, and can be expressed in cancer cells [54]. Some of them can be translated into proteins [55], [56], which show varying degrees of truncation and homology to the corresponding normal genes. Of eight Oct4 pseudogenes, six are Oct4A retro-pseudogenes [57], [58]. One of these may play a regulatory role in stem cells [59]. Eleven pseudogenes exist for Nanog [60]. While some of these Oct4 and Nanog pseudogenes can lead to protein products [56], others are transcribed but non-functional. For example, due to gene duplication the NanogP1 pseudogene has the same exon-intron structure as the Nanog gene, but a single nucleotide transition results in a premature stop codon [36]. This raises particular problems for RT-PCR analysis, as the approach used in many studies does not permit Oct4 and Nanog transcripts to be differentiated from non-functional pseudogene transcripts [35], [36]. Here we used a knockin approach to avoid these problems, and found that the Oct4 and Nanog genes are not transcribed in the melanoma and breast tumors studied. This observation is consistent with the findings of others. For example, expression of three Oct4 pseudogenes but not Oct4 itself has been reported in human breast tumors or gliomas [56]. These three pseudogenes gave rise to truncated protein products with a similar N-terminal transactivating domain as the bone fide Oct4 protein, and could be detected by anti-Oct4 antibodies. However, none of these truncated pseudogene-derived proteins exhibited Oct4-like activities [56].

In humans, the coding region of the Nanog-P8 pseudogene is 99.5% homologous to that of the Nanog gene, and is transcribed and translated into a protein that differs from the Nanog protein by only 3 amino acids [55]. It can be located in the nucleus and binds to the consensus Nanog DNA binding site. Expression of both Nanog and Nanog-P8 and the subcellular localization of the protein is cell type-specific [55]. Thus in humans, expression of the Nanog-P8 protein may functionally compensate for the absence of Nanog expression in a context-dependent manner. However, a pseudogene similar to Nanog-P8 has not been described in the mouse, excluding the possibility that a transcribed/translated pseudogene compensates for the lack of Nanog expression that we observed in the autochthonous cancer models used in our study.

Expression of Oct4 and Nanog in tumor cells grown as non-adherent spheres, conditions thought to enrich for CSCs, has been reported [20], [47], [48]. Furthermore, a recent study reported that cultivated MMTV-Neu cells express Nanog, and exhibit enhanced expression of Oct4 when grown as spheroids [61]. In contrast, we found no evidence for enhanced expression of Oct4 and Nanog upon the culturing of tumor cells either adherently or as spheroids. One possibility that we cannot rule out from our experiments is that long-term culturing of tumor cells may ultimately select for tumor cells that express Oct4 and/or Nanog. However, an alternative explanation could be that the reported expression of Oct4 and Nanog in these studies reflects pseudogene expression or another of the potential problems pointed out above.

In conclusion, in three independent autochthonous mouse models of melanoma and breast cancer we could find no evidence to support the notion that Oct4 and Nanog are expressed in the tumors, excluding the possibility that these transcriptional regulators determine the stemness properties of CSCs in these models. Although we cannot exclude that Oct4 and/or Nanog are expressed in CSCs and determine their properties in other types of cancer, this is certainly not the case in general. Moreover, given that pseudogene expression, the existence of alternatively-spliced isoforms and problems associated with antibody specificity complicate the analysis of Oct4 and Nanog expression, our data add further weight to the argument that care needs to be taken when assessing the role of Oct4 and Nanog in tumors and in particular in CSCs, and that conclusions made in the earlier literature may need to be reassessed.
